# Development and validation of a prediction model for infection in chronic nonhealing wounds: a two-center retrospective study with external validation

**DOI:** 10.3389/fpubh.2026.1813347

**Published:** 2026-05-19

**Authors:** Yang Jiang, Xingguo Nie, Hailong Feng, Guodong Wang, Xiugeng Li, Haijian Zhao, Jian Li

**Affiliations:** 1 Department of Burn Plastic Surgery and Medical Aesthetics, The First Affiliated Hospital of Henan Medical University, Weihui, Henan, China; 2 Department of Microsurgery of Hands and Feet, The First Affiliated Hospital of Henan Medical University, Weihui, Henan, China; 3 Department of Colorectal and Proctology, The First Affiliated Hospital of Henan Medical University, Weihui, Henan, China; 4 Department of Emergency Medicine, The First Affiliated Hospital of Henan Medical University, Weihui, Henan, China; 5 Department of Colorectal and Proctology, The First Affiliated Hospital of Henan Medical University, Weihui, Henan, China; 6 Department of Microsurgery of Hands and Feet, The First Affiliated Hospital of Henan Medical University, Weihui, Henan, China; 7 Burn Plastic Surgery and Medical Aesthetics Department, The First Affiliated Hospital of Henan Medical University, Weihui, Henan, China

**Keywords:** chronic nonhealing wounds, external validation, machine learning, two-center retrospective study, wound infection risk

## Abstract

**Purpose:**

To develop and externally validate a pragmatic and interpretable model that predicts infection risk in chronic nonhealing wounds using routine electronic medical record data.

**Methods:**

We conducted a two-center retrospective study at two tertiary hospitals in China. The primary cohort (*N =* 500) was split 7:3 into training (*N =* 350) and testing (*N =* 150) sets with stratified allocation; an external cohort (*N =* 300) was used for validation. Prespecified predictors were harmonized across sites. Feature selection used the overlap of Boruta and LASSO. Eight algorithms were compared with stratified five-fold cross-validation; the random forest (RF) was selected. Performance was assessed by AUROC with 95% CI, calibration, and decision curve analysis. Model interpretability was examined with SHAP.

**Results:**

Six consensus predictors were retained: smoking, diabetes duration, wound depth, elevated C-reactive protein, elevated procalcitonin, and hypoalbuminemia. The RF achieved AUROC 0.884 (95% CI 0.841–0.928) in the testing cohort with a calibration slope of 1.00 (95% CI 0.73–1.27) and higher net benefit than treat-all and treat-none across broad thresholds. External validation showed AUROC 0.855 (95% CI 0.807–0.904) with a calibration slope of 1.00 (95% CI 0.74–1.26) and similar decision utility. SHAP indicated hypoalbuminemia and inflammatory markers as dominant drivers, consistent with clinical reasoning.

**Conclusion:**

A six-variable RF model based on readily available data provides accurate, well-calibrated, and clinically useful prediction of infection in chronic nonhealing wounds, with transparent explanations to support bedside use. To facilitate immediate clinical application, this model has been deployed as a free, user-friendly web calculator. Prospective validation and impact evaluation across diverse settings are warranted.

## Introduction

Chronic nonhealing wounds are a substantial and growing public health problem, particularly among older adults and people with diabetes (1). When infection supervenes, the clinical trajectory often deteriorates, leading to prolonged hospitalization, repeated procedures, impaired quality of life, and increased costs (2). Timely recognition of patients at high risk for wound infection is therefore critical to guide debridement, antimicrobial therapy, and intensified wound care (3). In routine practice, however, diagnosis and risk stratification rely on heterogeneous clinical signs, delayed culture results, and clinician judgment that can vary across settings (4). Existing scoring tools are scarce, frequently derived from single centers, and often require data that are not consistently available at the bedside. As a result, clinicians lack a robust, generalizable instrument that transforms routinely collected information into an actionable estimate of infection risk.

Machine learning offers opportunities to improve prediction by modeling complex, nonlinear relationships among clinical features while retaining strong performance in heterogeneous populations (5). Nevertheless, many published models have important limitations, including small sample sizes (6), inadequate validation (7), and insufficient attention to calibration and clinical utility (8). Furthermore, the opacity of some machine learning methods can hinder adoption because users need transparent explanations that connect predictions to recognizable clinical patterns (9).

To address these gaps, we conducted a two-center, retrospective study to develop and validate a pragmatic prediction model for infection in chronic nonhealing wounds using variables that are routinely captured in electronic medical records. We prespecified candidate predictors based on clinical plausibility and prior literature, applied complementary feature selection strategies to identify a parsimonious set of robust variables, and compared multiple algorithms before selecting the best performing model. Model performance was evaluated comprehensively, including discrimination, calibration, and decision curve analysis, first in a held-out internal testing set and then in an independent external cohort from a second tertiary hospital. To enhance interpretability and support clinical trust, we used SHAP to quantify how each predictor contributes to individual and population-level risk estimates.

This study aims to deliver a transparent, externally validated tool that can assist bedside triage and early management of patients with chronic nonhealing wounds by identifying those at heightened risk of infection using information already available in routine care.

## Materials and methods

### Study design and participants

This two-center, retrospective cohort study was conducted at two tertiary hospitals in China. We identified a primary cohort of 500 consecutive patients with chronic nonhealing wounds managed at the First Affiliated Hospital of Henan Medical University between January 1, 2018, and June 30, 2025. Eligible patients were randomly assigned in a 7:3 ratio to the Training cohort (*N =* 350) and Testing cohort (*N =* 150). To preserve the event distribution, allocation was performed using stratified random sampling by infection status.

For external validation, we assembled an independent cohort of 300 patients meeting the same eligibility criteria at the First Central Hospital of Baoding during the same period. Data for both cohorts were obtained from the electronic medical records of the participating institutions.

Inclusion criteria were age ≥ 18 years; presence of a chronic nonhealing wound, defined as a wound persisting without complete epithelialization for at least 4 weeks despite standard care; an index encounter within the study period with sufficient documentation of baseline characteristics and wound status; and availability of clinical information adequate to ascertain wound infection status according to the predefined criteria used in this study.

Exclusion criteria were acute wounds of < 4 weeks’ duration; missing key predictor or outcome data that precluded model development or validation; duplicate records or repeated encounters of the same patient (only the first eligible encounter was retained); wounds primarily due to malignancy, radiation injury, or severe burns when these etiologies were the exclusive focus of care and not representative of chronic nonhealing wounds in routine practice; and patients transferred in or out without adequate baseline or outcome documentation.

This study adhered to the principles of the Declaration of Helsinki and was approved by the Institutional Review Boards of the First Affiliated Hospital of Henan Medical University and the First Central Hospital of Baoding. Given the retrospective design and use of de-identified data, the requirement for written informed consent was waived by the ethics committees.

### Identification of research variables

Candidate predictors were prespecified based on clinical plausibility, prior literature on chronic wound infection risk, and availability across both participating hospitals. Predictor definitions were standardized *a priori* and harmonized across sites to ensure consistency.

Demographic and admission variables included age (years) and sex (male/female), mode of admission (emergency, elective, or interhospital transfer), and body mass index (BMI, kg/m^2^) calculated from height and weight documented at the index encounter. Behavioral exposures comprised smoking status and alcohol consumption, each abstracted from the electronic medical record (EMR) and categorized as never versus previous/current use.

Comorbid conditions included diabetes mellitus (documented diagnosis or antidiabetic therapy in the EMR) with diabetes duration recorded in years, and hypertension (documented diagnosis or antihypertensive therapy). Clinical history variables included recent surgical history, captured from operative notes and discharge summaries as recorded in the EMR, and a history of prolonged bed rest as documented by treating clinicians or nursing notes prior to the index encounter.

Wound-related characteristics comprised wound depth and recent wound dressing changes. Wound depth was classified from wound care notes, surgical records, and/or imaging as deep dermal involvement versus full-thickness (exposure of subcutaneous tissue, fascia, muscle, tendon, or bone). Recent wound dressing changes reflected whether dressing change activity was documented in the EMR at or immediately preceding the index encounter.

Baseline laboratory biomarkers were abstracted from the index date; if multiple measurements were available, the result closest in time to the index assessment was used. Binary laboratory indicators were defined relative to each institution’s laboratory reference intervals: elevated C-reactive protein (CRP), elevated erythrocyte sedimentation rate (ESR), elevated procalcitonin (PCT), and elevated white blood cell count (WBC) were coded as “elevated” when above the local upper limit of normal. Hypoalbuminemia and decreased hemoglobin (anemia) were coded as “yes” when below the local lower limit of normal. This laboratory-based approach ensured comparability despite potential inter-laboratory differences in assay methods and reference ranges.

All predictor variables were derived from data available at or before the index encounter to prevent information leakage. Continuous variables were retained in their native scales, and categorical variables were coded as indicated in Table 1. Where necessary, polytomous variables were represented with mutually exclusive categories for model building.

**Table 1:** Baseline characteristics of the training, testing, and external validation sets.

Characteristic	Training cohort *N =* 350	Testing cohort *N =* 150	Validation cohort *N =* 300	*P*
Age(years), mean ± SD	63.1 ± 13.0	61.0 ± 12.4	64.2 ± 12.8	0.18
Sex, *n* (%)				0.09
Male	222 (63.4)	106 (70.7)	170 (56.7)	
Female	128 (36.6)	44 (29.3)	130 (43.3)	
Mode of admission, *n* (%)				0.36
Emergency	170 (48.6)	62 (41.3)	130 (43.3)	
Elective	120 (34.3)	57 (38.0)	105 (35.0)	
Transfer	60 (17.1)	31 (20.7)	65 (21.7)	
BMI (kg/m^2^), mean ± SD	25.9 ± 4.1	25.2 ± 3.7	26.1 ± 3.9	0.24
Recent surgical history, *n* (%)				0.19
No	280 (80.0)	128 (85.3)	237 (79.0)	
Yes	70 (20.0)	22 (14.7)	63 (21.0)	
Recent wound dressing changes, *n* (%)				0.12
No	195 (55.7)	97 (64.7)	171 (57.0)	
Yes	155 (44.3)	53 (35.3)	129 (43.0)	
Alcohol consumption, *n* (%)				0.09
Never	233 (66.6)	94 (62.7)	222 (74.0)	
Previous/Current	117 (33.4)	56 (37.3)	78 (26.0)	
Smoking, *n* (%)				0.10
Never	220 (62.9)	108 (72.0)	180 (60.0)	
Previous/Current	130 (37.1)	42 (28.0)	120 (40.0)	
Diabetes mellitus, *n* (%)				0.29
No	104 (29.7)	52 (34.7)	102 (34.0)	
Yes	246 (70.3)	98 (65.3)	198 (66.0)	
Diabetes duration (years), M (Q1, Q3)	8.5 (4.0, 14.0)	7.0 (4.0, 12.0)	9.0 (5.0, 15.0)	0.09
Hypertension				0.20
No	144 (41.1)	74 (49.3)	138 (46.0)	
Yes	206 (58.9)	76 (50.7)	162 (54.0)	
Wound depth				0.08
Deep dermal involvement	196 (56.0)	75 (50.0)	192 (64.0)	
Full-thickness	154 (44.0)	75 (50.0)	108 (36.0)	
History of prolonged bed rest				0.11
No	258 (73.7)	102 (68.0)	234 (78.0)	
Yes	92 (26.3)	48 (32.0)	66 (22.0)	
Elevated C-reactive protein				0.07
No	180 (51.4)	69 (46.0)	183 (61.0)	
Yes	170 (48.6)	81 (54.0)	117 (39.0)	
Elevated ESR				0.18
No	164 (46.9)	83 (55.3)	135 (45.0)	
Yes	186 (53.1)	67 (44.7)	165 (55.0)	
Elevated PCT				0.12
No	230 (65.7)	112 (74.7)	198 (66.0)	
Yes	120 (34.3)	38 (25.3)	102 (34.0)	
Elevated WBC				0.17
No	212 (60.6)	105 (70.0)	192 (64.0)	
Yes	138 (39.4)	45 (30.0)	108 (36.0)	
Hypoalbuminemia				0.09
No	240 (68.6)	114 (76.0)	225 (75.0)	
Yes	110 (31.4)	36 (24.0)	75 (25.0)	
Decreased hemoglobin				0.07
No	200 (57.1)	95 (63.3)	201 (67.0)	
Yes	150 (42.9)	55 (36.7)	99 (33.0)	
Wound infection, *n* (%)	78 (22.3)	40 (26.7)	51 (17.0)	0.08

Data are presented as mean ± standard deviation (SD) for continuous variables, *n* (%) for categorical variables, and median (Q1, Q3) for non-normally distributed variables. *P*-values indicate comparisons among the training, testing, and external validation cohorts. BMI, Body Mass Index; CRP, C-reactive protein; ESR, Erythrocyte Sedimentation Rate; PCT, Procalcitonin; WBC, White Blood Cell; Q1, 25th percentile; Q3, 75th percentile.

### Outcome definition and ascertainment

Wound infection was operationally defined based on International Wound Infection Institute criteria and electronic medical record documentation within 14 days of the index encounter. Diagnosis required the presence of clinical signs of local infection, including purulent discharge, spreading erythema, warmth, swelling, increasing pain, foul odor, or wound breakdown, or evidence of positive wound cultures accompanied by targeted antimicrobial therapy or surgical debridement. Outcomes were retrospectively ascertained from physician notes, wound assessments, and prescription records. To ensure reproducibility and minimize bias, two blinded clinical researchers independently reviewed the data, with any discrepancies resolved by consensus with a third senior wound care specialist. Consistent with the TRIPOD statement, our model is conceptualized as a diagnostic prediction model, estimating the concurrent or imminent probability of infection within this 14-day horizon.

To ensure the clinical relevance of the outcome, a definitive diagnosis of wound infection in our study required both documented local clinical signs and subsequent targeted therapeutic intervention, such as the initiation or escalation of systemic antibiotics or surgical wound debridement. Microbiological culture results were available and supportive in approximately 68% of the adjudicated infection cases; however, cases meeting the clinical and treatment criteria were still classified as infected even if cultures were not obtained prior to empirical treatment. Furthermore, to minimize bias during the retrospective chart review, the adjudicators evaluating the infection outcomes were instructed to base their decisions strictly on wound care nursing records, physician progress notes, and microbiology reports, effectively blinding them to systemic biomarker profiles during the outcome adjudication phase.

### Feature selection

We used two complementary methods on the same preprocessed data: Boruta (a random-forest wrapper that creates shuffled “shadow” versions of each variable and keeps a variable only if it consistently beats its shadows; Bonferroni *α* = 0.05; up to 100 iterations with tentative variables resolved by the rough-fix rule) and LASSO logistic regression (adds an L1 penalty that shrinks unhelpful coefficients to zero; 10-fold stratified cross-validation with the 1-SE rule to pick *λ*). The final predictor set was the overlap between Boruta-confirmed variables and non-zero LASSO coefficients, prioritizing features that are both robust and simple. To avoid information leakage, all preprocessing steps were fitted on the training data and then applied unchanged to the testing and external validation sets.

### Model construction and comparison

Following feature selection, only the consensus predictors retained by both Boruta and LASSO were used to build eight models: decision tree, k-nearest neighbors, Light Gradient Boosting Machine, naïve Bayes, random forest, support vector machine, extreme gradient boosting, and multivariable logistic regression. All modeling was performed on the training cohort; the internal testing set and the external validation cohort were reserved for evaluation only.

Hyperparameters were tuned within the stratified five-fold cross-validation framework using grid search optimization. To ensure full transparency and reproducibility, the specific hyperparameter configurations for the final selected Random Forest model are comprehensively detailed in Supplementary Table S3. Model ranking prioritized the mean cross-validated area under the receiver operating characteristic curve (AUROC); accuracy, sensitivity, specificity, precision/recall, F1 score, and the area under the precision–recall curve were summarized as secondary measures. After tuning, a final instance of each algorithm was refit on the full training cohort with the selected hyperparameters.

Generalizability was assessed in two stages: first on the held-out testing set, then on the external validation cohort. For both datasets, we reported AUROC with 95% confidence intervals and threshold-dependent metrics at a prespecified operating point chosen by maximizing Youden’s index on the training data. Calibration was examined using reliability curves together with calibration intercept and slope, and clinical utility was appraised by decision curve analysis across clinically relevant thresholds.

### The SHAP to model interpretation

We used SHAP (Shapley Additive Explanations) to make the model’s decisions understandable. In short, SHAP shows how each predictor pushes the predicted risk up or down for each individual (local explanation) and how important each predictor is on average across all individuals (global explanation). SHAP values were calculated on the model’s logit (log-odds) scale; when a probability view was needed, we converted the contributions to the probability scale for easier interpretation. Global importance was summarized by the average absolute SHAP value per variable.

**Figure 1:**
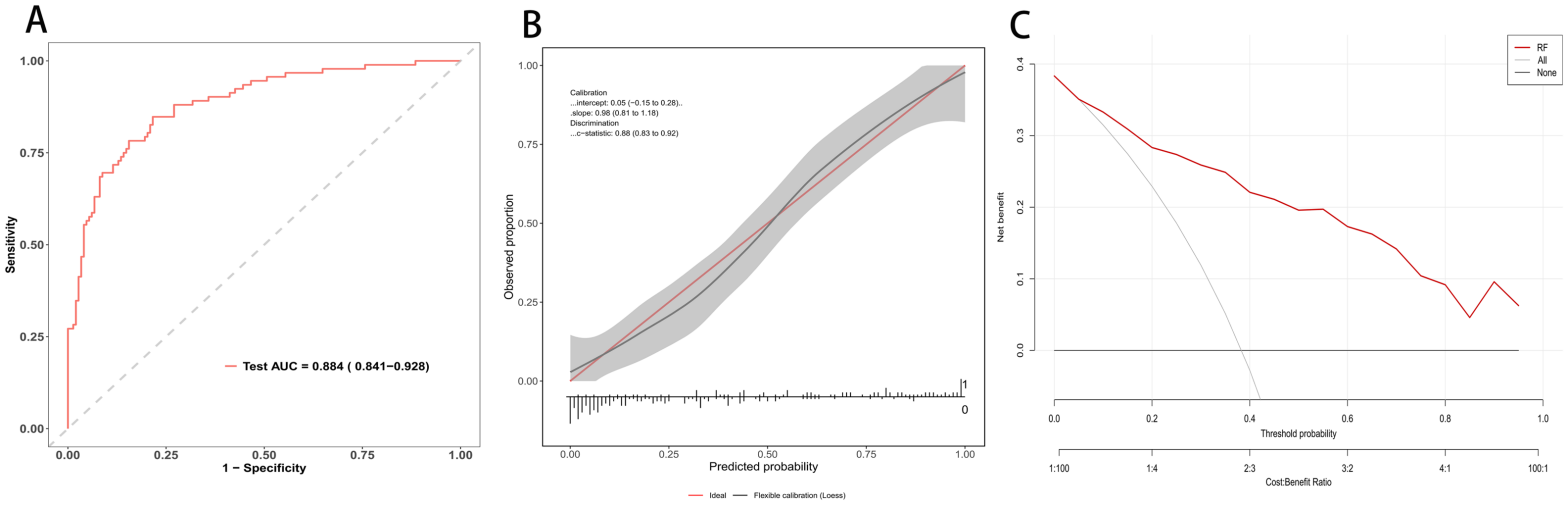
RF model evaluation in testing cohort. **(A)** ROC curve on the independent test set; AUC = 0.884 (95% CI 0.841–0.928). **(B)** Calibration plot with flexible curve and 95% CI band versus the ideal line; calibration intercept, slope, and c-statistic are shown. **(C)** Decision curve analysis: net benefit across threshold probabilities for the random forest (red) compared with treat-all and treat-none strategies.

Figure 1A shows a simple bar chart: longer bars mean the variable has a larger overall impact on predictions. Figure 1B shows a beeswarm plot: each dot is a patient; color reflects the original feature value (low to high), and the position on the x-axis shows whether that feature increased (right) or decreased (left) the predicted risk.

### Statistical analyses

All statistical analyses and data visualizations were performed using.

R (version 4.4.2) and JD_DCPM (V6.11, Jingding Medical Technology Co., Ltd.). Continuous variables were assessed for normality via the Shapiro–Wilk test. Normally distributed data are presented as mean ± standard deviation, with group comparisons conducted using Student’s *t*-tests. Non-normally distributed variables are expressed as median and interquartile range [M (Q1, Q3)] and analyzed via the Mann–Whitney *U* test. Categorical variables are reported as frequencies (percentages) and evaluated using Chi-square tests or Fisher’s exact tests (for cell counts <5). Statistical significance was defined as a two-tailed *p* < 0.05.

To address missing data, a complete-case analysis was primarily utilized for model development and validation. This strategy was justified because the overall proportion of missing values for key predictors was prospectively evaluated and found to be exceptionally low, minimizing the risk of significant bias. To rigorously verify this assumption and align with robust methodological standards, a sensitivity analysis was performed using multiple imputation by chained equations. Missing values were imputed to create five complete datasets. The predictive models were then refitted and evaluated on these imputed datasets, allowing us to compare the pooled performance metrics with those derived from our primary complete-case analysis. To justify the dichotomization of continuous inflammatory markers and assess potential information loss, a sensitivity analysis was conducted. We retrained the best-performing model using the continuous representations of these variables and compared its predictive performance to the primary binarized model.

## Result

### Patient characteristics for training, testing, and external validation cohorts

Table 1 summarizes baseline characteristics for the training (*N =* 350), testing (*N =* 150), and external validation (*N =* 300) cohorts. Participants were predominantly older adults (mean age 61–64 years) with BMI near 26 kg/m^2^; males constituted approximately 57–71%. The distributions of admission pathways, behavioral exposures (tobacco and alcohol), and comorbidities were comparable across cohorts; diabetes was present in about two-thirds of participants (median duration 7–9 years), and hypertension in roughly one-half. Wound-related features (depth) and recent care exposures (surgical history, dressing changes, prolonged bed rest) were similarly distributed.

Laboratory indices, including CRP, ESR, PCT, and WBC counts as well as serum albumin and hemoglobin levels, varied within expected ranges with no systematic differences between cohorts. Crude wound infection rates ranged from 17 to 27% and did not differ significantly among cohorts.

While statistical testing did not show extreme deviations in several variables, clinically noticeable variations were observed across the cohorts. Most notably, there were observable differences in sex distribution, the proportion of full-thickness wounds, baseline inflammatory markers, hypoalbuminemia rates, and overall infection prevalence. Rather than indicating a perfectly balanced uniform population, these variations represent expected real-world case-mix differences between independent clinical centers.

The detailed patient screening and cohort assembly process is illustrated in Supplementary Figure 1. Initially, 526 patients were assessed for eligibility in the primary cohort, of which 26 patients (4.9%) were excluded due to missing key predictor data (predominantly laboratory markers such as procalcitonin and C-reactive protein). The remaining 500 eligible patients were subsequently stratified and randomly split in a 7:3 ratio into the training cohort (*N =* 350, with 78 infection events [22.3%]) and the testing cohort (*N =* 150, with 40 infection events [26.7%]). Similarly, for the external validation cohort, 315 patients were initially evaluated, and 15 patients (4.8%) were excluded due to missing data, resulting in a final validation cohort of 300 patients (51 infection events [17.0%]). Because the overall missingness was exceptionally low across both centers, a complete-case analysis was justified as the primary handling strategy. In our sensitivity analysis utilizing multiple imputation (MICE) for these missing values, the refitted model yielded predictive performance metrics nearly identical to the complete-case analysis (AUROC difference < 0.01). This confirms that our primary handling strategy was robust and did not introduce measurable bias.

### Feature selection

**Figure 2:**
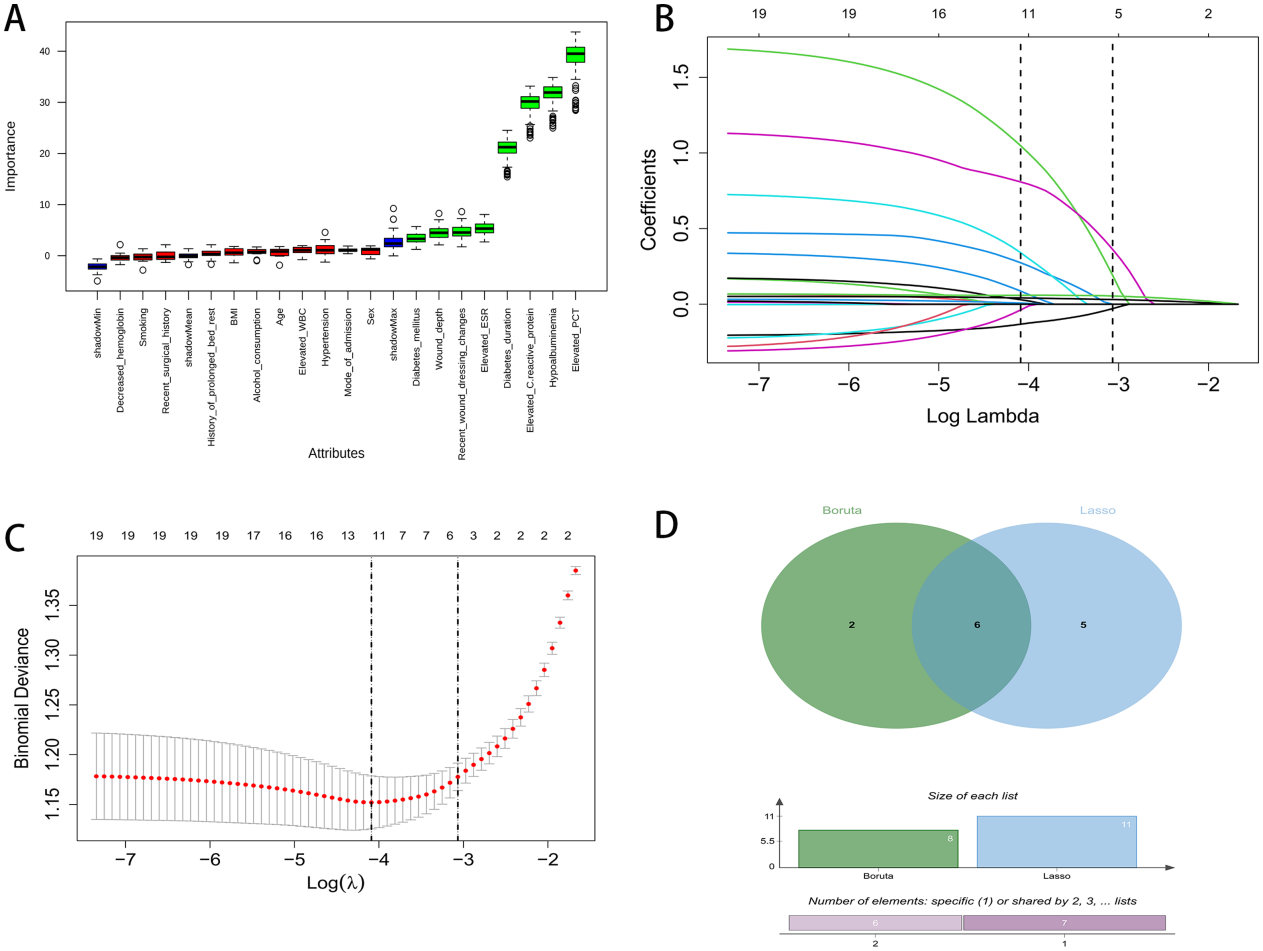
Feature selection process using Boruta and LASSO regression. **(A)** Boruta variable importance plot. **(B)** LASSO coefficient paths. **(C)** LASSO cross-validation curve. **(D)** Venn diagram of Boruta and LASSO. The overlapping region represents the 6 key features retained for the final model. Labels indicate set sizes: green (Boruta-selected: 8 features), blue (LASSO-selected: 11 features), overlap (shared: 6 features).

We applied two complementary algorithms, Boruta and LASSO logistic regression, to the preprocessed training dataset. Boruta confirmed eight candidate predictors, and LASSO with 10-fold cross-validation using the one-standard-error criterion retained 11 predictors with nonzero coefficients. The intersection yielded six consensus features: Smoking, Diabetes duration, Wound depth, Elevated C-reactive protein, Elevated PCT, and Hypoalbuminemia. All preprocessing and selection steps were performed in the training cohort and then applied unchanged to the testing and external validation cohorts to avoid information leakage. These six variables were carried forward for model development and evaluation (Figure 2).

### Model development and performance

**Figure 3:**
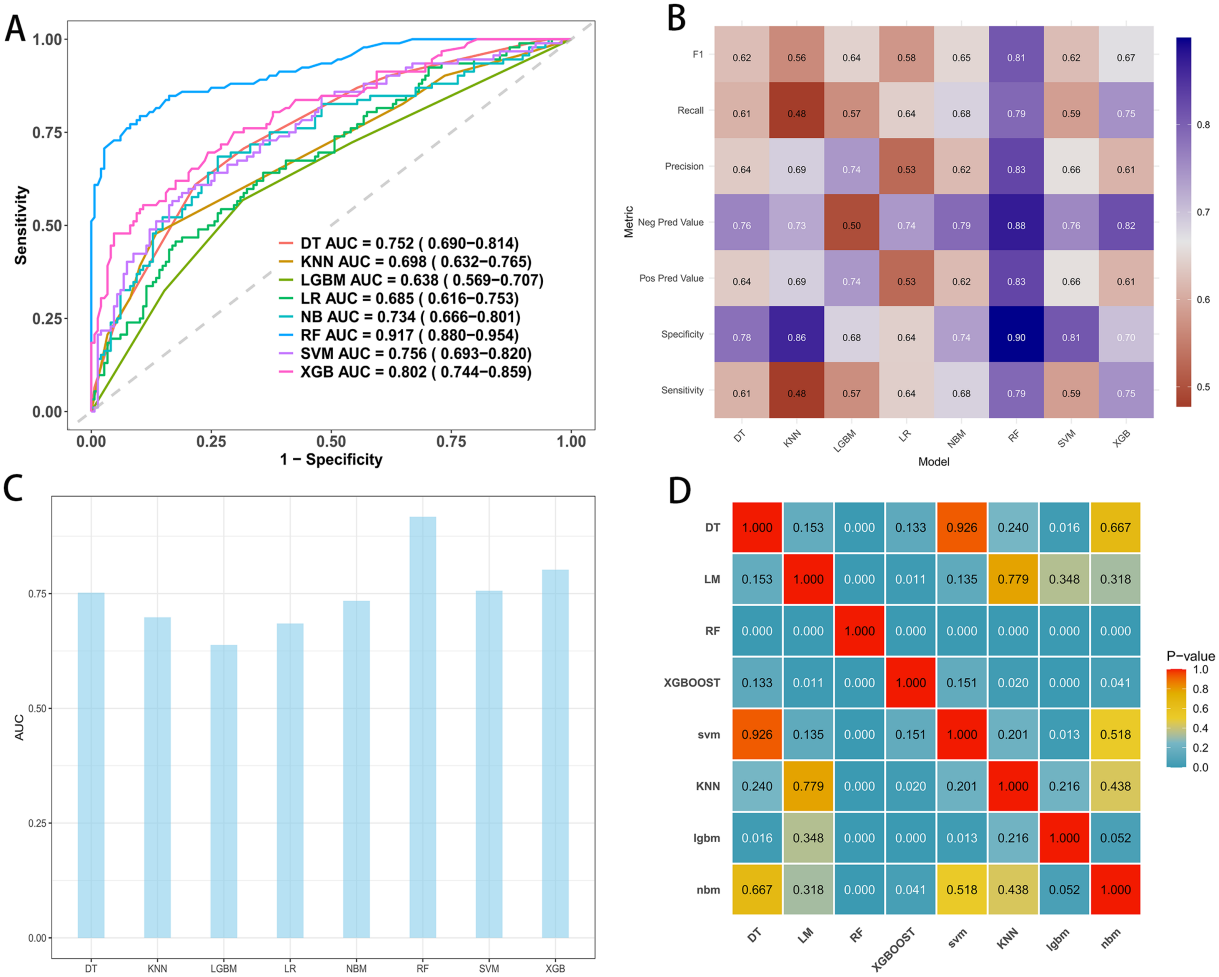
Performance metrics of models in the training cohort. **(A)** ROC curves and AUC values for the eight model. **(B)** Comparative analysis metrics across all eight models. **(C)** Bar chart summarizing AUROC by model. **(D)** Pairwise AUROC comparisons using the DeLong test; cells show *p*-values.

Using the six selected predictors, we trained eight algorithms in the training cohort with stratified five-fold cross-validation and hyperparameter tuning. As shown in Figures 3A–C, the random forest achieved the highest discrimination with an AUROC of 0.917 (95% CI 0.880–0.954), followed by extreme gradient boosting at 0.802 (0.744–0.859), support vector machine at 0.756 (0.693–0.820), decision tree at 0.752 (0.690–0.814), naïve Bayes at 0.734 (0.666–0.801), k-nearest neighbors at 0.698 (0.632–0.765), logistic regression at 0.685 (0.616–0.753), and LightGBM at 0.638 (0.569–0.707). Secondary metrics summarized in Figure 3B were concordant with the AUROC ranking, indicating that the random forest provided the most favorable balance of sensitivity and specificity. Pairwise AUROC comparisons using the DeLong test (Figure 3D) supported the superiority of the random forest over most comparators, with several differences reaching statistical significance. Based on these results, the random forest was selected as the primary model for subsequent testing and external validation.

### Model performance on both the testing and external validation sets

In the independent testing cohort, the random forest achieved strong discrimination with an AUROC of 0.884 (95% CI 0.841 to 0.928) as shown in Figure 1A. Calibration was well aligned with the ideal reference (Figure 1B), with a calibration slope of 0.98 (95% CI 0.81 to 1.18), Brier scores of 0.14 (95% CI: 0.11–0.18), and a c statistic of 0.88 (95% CI 0.83 to 0.92). Decision curve analysis indicated higher net benefit than treat-all and treat-none across a wide range of threshold probabilities, with the largest advantage at lower thresholds (Figure 1C).

**Figure 4:**
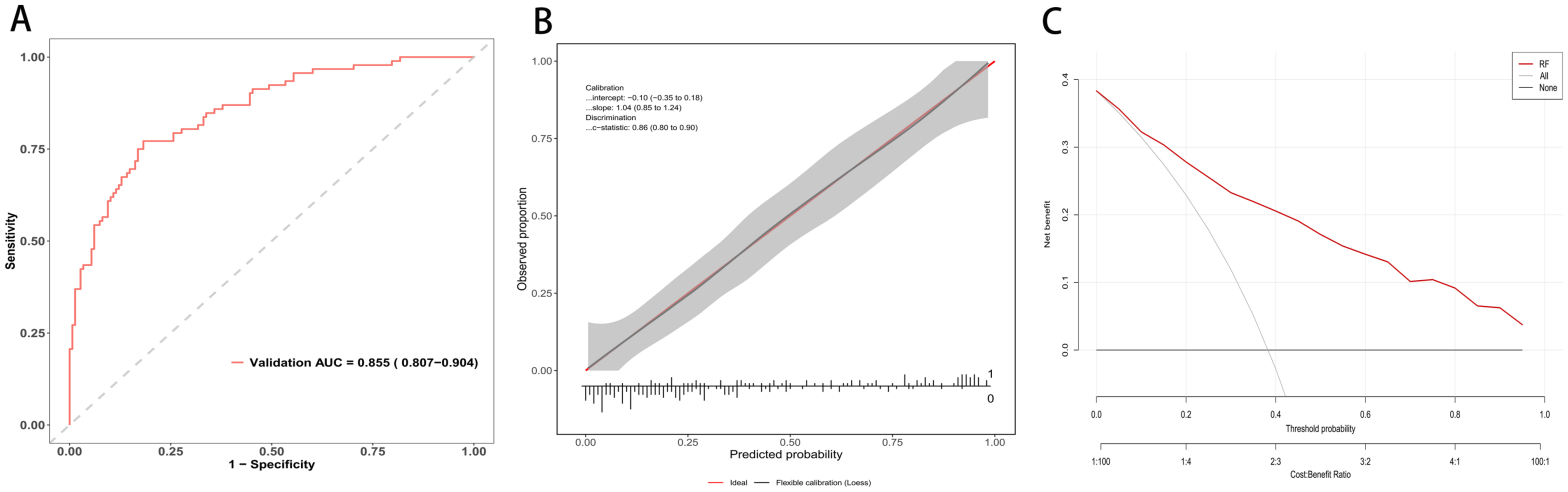
RF model evaluation in external validation cohort. **(A)** ROC curve in the validation cohort; AUC = 0.855 (95% CI 0.807–0.904). **(B)** Calibration curve of the external validation set. **(C)** Decision curve analysis: net benefit across threshold probabilities for the model (red) vs. treat-all and treat-none.

In the external validation cohort, the random forest preserved a strong discrimination with an AUROC of 0.855 (95% CI 0.807–0.904) as shown in Figure 4A. Calibration was close to ideal (Figure 4B), with a calibration slope of 1.04 (95% CI 0.85 to 1.24), Brier scores of 0.15 (95% CI: 0.11–0.20), and a c-statistic of 0.86 (95% CI 0.80 to 0.90). Decision curve analysis demonstrated a consistent net benefit across a broad range of threshold probabilities compared with treat-all and treat-none strategies (Figure 4C).

Furthermore, a sensitivity analysis was performed to evaluate the impact of binarizing continuous inflammatory markers. When utilizing the continuous representations of C-reactive protein and procalcitonin, the Random Forest model achieved an AUROC of 0.895 in the testing cohort and 0.862 in the external validation cohort. These performances were only marginally higher than those of the primary binarized model (AUROC = 0.884 and 0.855, respectively). Detailed comparisons are provided in Supplementary Table S4. Consequently, the binarized model was retained as the primary tool to maintain alignment with standard clinical laboratory thresholds and enhance bedside interpretability.

Additionally, to provide empirical insight into the model’s stability across different etiologies, an exploratory descriptive summary of model performance by major wound categories in the validation cohort is presented in Supplementary Table S5. While the AUROC values remained consistent across subgroups, the wide confidence intervals due to limited subgroup sample sizes warrant cautious interpretation.

### Model interpretability

**Figure 5:**
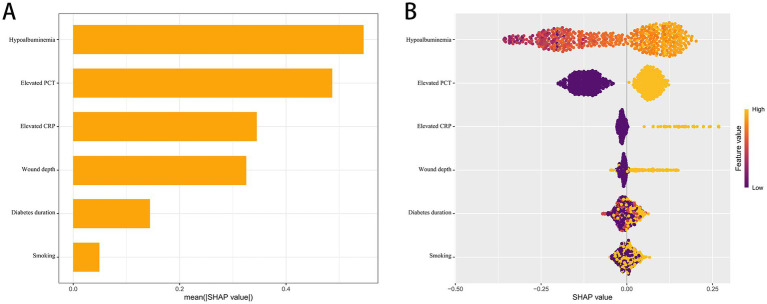
SHAP analysis of the RF model. **(A)** Global importance ranked by mean absolute SHAP value. **(B)** Beeswarm of per-patient SHA*p* values for key predictors.

Model interpretability was assessed using SHAP, with global importance (Figure 5A) ranking the predictors by their mean absolute SHAP values. The most influential variable was hypoalbuminemia, followed by elevated procalcitonin (PCT), elevated C-reactive protein (CRP), wound depth, diabetes duration, and smoking. The summary beeswarm plot (Figure 5B) further illustrates the effects of these features, revealing that higher values or the presence of each variable consistently increases predicted risk. Specifically, patients with hypoalbuminemia, elevated PCT or CRP, greater wound depth, longer diabetes duration, and a history of smoking are all shifted toward higher probabilities of the adverse outcome according to the model. The color gradient underscores this positive association: red (representing higher values or presence) tends to cluster on the right, corresponding to positive SHAP values that drive predictions upward, while blue (lower values or absence) lies to the left. These results confirm that all six variables act as positive drivers of risk in this model, and the SHAP analysis provides transparent, patient-level explanations that are consistent with clinical understanding, highlighting the central role of inflammation, poor nutritional status, tissue severity, duration of diabetes, and smoking exposure in elevating the likelihood of the outcome.

To directly translate our best-performing Random Forest model into an accessible clinical tool, we developed a free, interactive web-based calculator (freely accessible at: https://my-portfolio.shinyapps.io/Chronic_Nonhealing_Wound_Infection_Predictor/). By simply inputting the six selected predictors at the bedside, clinicians can instantly obtain an individualized probability of wound infection. Furthermore, the calculator automatically stratifies patients into low (< 20%), moderate (20–50%), or high (> 50%) risk categories, offering concrete threshold recommendations to guide targeted clinical interventions.

## Discussion

This two-center retrospective study developed and validated a bedside model to predict infection in chronic nonhealing wounds using routine electronic medical record data from two tertiary hospitals in China. After comparing eight algorithms, the random forest model performed best. It achieved an AUROC of 0.884 in the internal testing cohort and 0.855 in the external cohort. Calibration was close to ideal in both cohorts, and decision curve analysis showed consistent net benefit across a wide range of thresholds. These results compare well with prior studies in diabetic foot infection and other chronic wounds, where models often originated from a single center, used larger and less practical variable sets, and did not report calibration or clinical utility.

The final set of six predictors reflects clear clinical mechanisms and is easy to collect at the bedside. Hypoalbuminemia contributed the most to predicted risk, which fits the link between poor nutrition, weaker immune defense, and slower tissue repair (10). Elevated procalcitonin and C-reactive protein captured systemic inflammatory burden that often accompanies bacterial infection in chronic wounds (11, 12). Greater wound depth signaled more severe tissue injury and likely higher bacterial load and ischemic risk (13, 14). Longer diabetes duration and smoking reflected chronic microvascular damage and impaired host defenses (15, 16). SHAP values showed that each variable increased risk in a way that matches clinical reasoning and provided patient-level explanations that can support communication and shared decisions. The absence of white blood cell count and erythrocyte sedimentation rate from the final model is consistent with reports that C-reactive protein and procalcitonin often show stronger and more specific links with infection in chronic ulcers (17).

This work adds to the field by using predefined and harmonized predictors, two complementary feature selection methods to reduce overfitting, and a transparent evaluation that includes discrimination, calibration, and decision curves together with model explanations (18). The small and practical predictor set improves usability and supports integration into clinical workflows (19). In practice, the model can help identify high-risk patients early, guide closer monitoring, support timely debridement, inform antimicrobial decisions, and prompt supportive measures such as nutrition support, improved diabetes control, and smoking cessation (20). Because all variables are standard in many hospitals, deployment should not require new tests or devices. A common criticism of machine learning models is the lack of parsimony and the inherent difficulty of translating complex algorithms into bedside practice. While a simple Logistic Regression model would be highly parsimonious, our results demonstrated that it achieved an inadequate AUROC of 0.685, rendering it insufficient for accurate clinical decision-making. To justify the use of the more complex Random Forest model and ensure its clinical utility, we deployed it as a user-friendly, web-based calculator. This digital tool circumvents the need for manual calculations or complex nomograms, allowing clinicians to input routine patient data and instantly obtain individualized risk probabilities. This strategy effectively harmonizes algorithmic complexity with practical clinical application. A critical consideration when deploying advanced machine learning algorithms is whether their complex, “black-box” nature is justified by their actual clinical utility. Based on our Decision Curve Analysis (DCA), the Random Forest model demonstrated a substantial and consistent increase in net benefit across a wide range of threshold probabilities compared to both default strategies (treat-all or treat-none) and the simpler Logistic Regression model. In real-world clinical settings, this superior net benefit translates directly into optimized patient care: it maximizes the early detection of high-risk patients requiring immediate intervention while safely preventing unnecessary treatments and antibiotic misuse in low-risk individuals. We argue that this profound improvement in patient outcomes overwhelmingly justifies the trade-off of reduced algorithmic transparency. Furthermore, by translating this complex model into an accessible web-based calculator and providing clear feature importance rankings, we have successfully mitigated the interpretability barrier, empowering clinicians to leverage high-level predictive accuracy through a simplified, user-friendly interface.

To facilitate the translation of our predictive model into routine clinical practice, we developed an interactive, web-based risk calculator, which is freely accessible at: https://my-portfolio.shinyapps.io/chronic_nonhealing_wound_infection_predictor/. This point-of-care tool allows clinicians to input six readily available patient parameters, including smoking history, diabetes duration, wound depth, and systemic inflammatory and nutritional markers (CRP, PCT, and albumin), to instantly compute an individualized probability of wound infection. Based on the output, patients are dynamically stratified into low, moderate, or high-risk categories. This real-time risk stratification enables physicians to implement timely and targeted interventions, such as closer wound monitoring, early empirical antibiotic therapy, or aggressive debridement for high-risk individuals. Furthermore, the underlying algorithm of this model is lightweight and highly adaptable, presenting a clear practical pathway for future integration into hospital Electronic Health Record (EHR) systems. Such integration would allow for automated, real-time risk scoring upon admission or during outpatient follow-ups, seamlessly enhancing clinical decision-making without adding to the cognitive burden of healthcare providers.

This study has several limitations. A notable methodological consideration in our study is the potential risk of incorporation bias. Because elevated systemic inflammatory markers often trigger a clinician’s suspicion of infection, there is a risk that these predictors might be intrinsically linked to the outcome definition. We mitigated this risk by strictly adhering to the International Wound Infection Institute criteria for our outcome adjudication, which heavily prioritize local wound characteristics rather than systemic criteria. Additionally, our outcome adjudicators evaluated infection status independently of the systemic laboratory flowsheets. Consequently, the laboratory biomarkers served as true independent predictors of a locally defined event rather than artifacts of the diagnostic criteria itself. The retrospective design can introduce unmeasured confounding and some misclassification of infection despite predefined criteria. Laboratory markers were binarized by local reference ranges to improve portability, which may reduce information from continuous values. Because our cohorts were derived from two hospitals in China, the generalizability of the model to other geographic regions, different levels of care, and diverse wound etiologies requires further validation. Some potentially useful data were not included, such as wound area and its change over time, limb perfusion, long-term glycemic control, imaging features, and microbiology. Furthermore, due to the limited overall sample size, formal and statistically robust subgroup comparisons were underpowered. Although we have provided an exploratory descriptive summary of model performance across major wound categories to offer empirical insights, the small number of infection events in specific subgroups resulted in wide confidence intervals. Therefore, these subgroup-level metrics must be interpreted cautiously. Future large-scale, multicenter studies with expanded cohorts are required to further validate our findings and conduct detailed subgroup investigations. We also did not measure clinical impact, so effects on decision making, healing time, antibiotic use, and length of stay remain unknown. Future work should include prospective multicenter validation with model updating, evaluation in varied care settings and patient groups, inclusion of richer predictors and continuous lab values, monitoring for performance over time, and impact studies that compare model-guided care with usual care. Fairness across subgroups and selection of action-specific thresholds should also be evaluated.

## Conclusion

This study presents a practical and transparent model that predicts infection in chronic nonhealing wounds using routine clinical data. The model demonstrated strong discrimination, reliable calibration, and consistent clinical net benefit across both internal and external validation cohorts. The small, clinically coherent predictor set and SHAP-based explanations support bedside use and clear communication, which is further empowered by the development of our free web-based calculator (https://my-portfolio.shinyapps.io/Chronic_Nonhealing_Wound_Infection_Predictor/). Although the retrospective design, binarized laboratory inputs, and two-center setting limit generalizability and leave clinical impact untested, the work provides a solid foundation for prospective validation, model updating across diverse sites, and implementation studies in real workflows. If future studies confirm performance and utility, this tool could facilitate the early identification of high-risk patients, support timely interventions, strengthen nutritional and metabolic management, and improve patient outcomes while promoting responsible antibiotic use.

## Data Availability

The raw data supporting the conclusions of this article will be made available by the authors, without undue reservation.
