# Supplementary material for: Development and validation of a prediction model for infection in chronic nonhealing wounds: a two-center retrospective study with external validation
Source: Front Public Health. 2026 May 19;14:1813347. doi: 10.3389/fpubh.2026.1813347 (PMC13226498; doi:10.3389/fpubh.2026.1813347)

**Supplementary Figure 1.** Study flowchart detailing the patient screening, exclusion, and cohort assembly process.


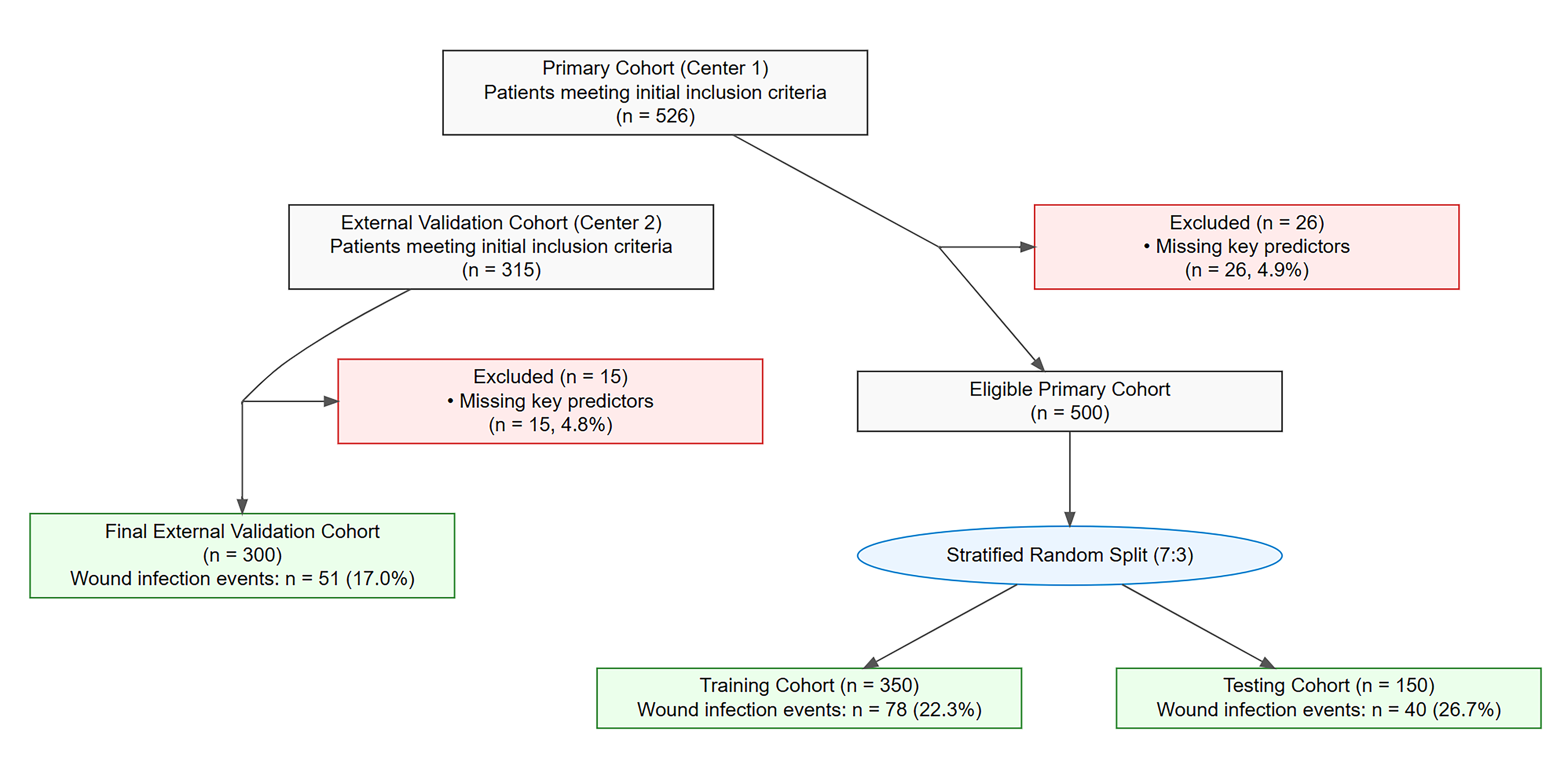

Supplement: Supplementary file 1 [file Supplementary_file_1.docx]
