# Supplementary material for: Development and validation of a prediction model for infection in chronic nonhealing wounds: a two-center retrospective study with external validation
Source: Front Public Health. 2026 May 19;14:1813347. doi: 10.3389/fpubh.2026.1813347 (PMC13226498; doi:10.3389/fpubh.2026.1813347)
Supplement: Supplementary file 2 [file Table_1.docx]

Supplementary Table S1. Distribution of missing data among the initial extracted cohorts prior to complete-case exclusion

| **Characteristic** | **Initial Training cohort (N=368)** | **Initial Testing cohort (N=158)** | **Initial Validation cohort (N=315)** | **P value** |
| --- | --- | --- | --- | --- |
| Age | 0 (0.0%) | 0 (0.0%) | 0 (0.0%) | >0.99 |
| Sex | 0 (0.0%) | 0 (0.0%) | 0 (0.0%) | >0.99 |
| Mode of admission | 0 (0.0%) | 0 (0.0%) | 0 (0.0%) | >0.99 |
| BMI | 4 (1.1%) | 1 (0.6%) | 2 (0.6%) | 0.72 |
| Recent surgical history | 0 (0.0%) | 0 (0.0%) | 0 (0.0%) | >0.99 |
| Recent wound dressing changes | 0 (0.0%) | 0 (0.0%) | 0 (0.0%) | >0.99 |
| Alcohol consumption | 2 (0.5%) | 0 (0.0%) | 1 (0.3%) | 0.58 |
| Smoking | 2 (0.5%) | 1 (0.6%) | 2 (0.6%) | 0.95 |
| Diabetes mellitus | 0 (0.0%) | 0 (0.0%) | 0 (0.0%) | >0.99 |
| Diabetes duration* | 6 (1.6%) | 2 (1.3%) | 5 (1.6%) | 0.96 |
| Hypertension | 0 (0.0%) | 0 (0.0%) | 0 (0.0%) | >0.99 |
| Wound depth | 3 (0.8%) | 1 (0.6%) | 2 (0.6%) | 0.91 |
| History of prolonged bed rest | 0 (0.0%) | 0 (0.0%) | 0 (0.0%) | >0.99 |
| Elevated C-reactive protein | 11 (3.0%) | 5 (3.2%) | 8 (2.5%) | 0.89 |
| Elevated ESR | 9 (2.4%) | 4 (2.5%) | 7 (2.2%) | 0.96 |
| Elevated PCT | 14 (3.8%) | 6 (3.8%) | 12 (3.8%) | >0.99 |
| Elevated WBC | 2 (0.5%) | 1 (0.6%) | 2 (0.6%) | 0.95 |
| Hypoalbuminemia | 5 (1.4%) | 2 (1.3%) | 6 (1.9%) | 0.83 |
| Decreased hemoglobin | 4 (1.1%) | 2 (1.3%) | 5 (1.6%) | 0.86 |
| Wound infection (Outcome) | 0 (0.0%) | 0 (0.0%) | 0 (0.0%) | >0.99 |
| **Total excluded cases** | **18 (4.9%)** | **8 (5.1%)** | **15 (4.8%)** | **0.98** |

*Note: Missing percentages are calculated based on the total initial number of patients extracted for each cohort. Cases with any missing key predictor were excluded to form the final study cohorts (Training N=350, Testing N=150, Validation N=300). P values indicate the comparison of missingness rates across the three cohorts using the Chi-square test or Fisher's exact test, showing no significant difference in missing data distribution.*
