# Supplementary material for: Development and validation of a prediction model for infection in chronic nonhealing wounds: a two-center retrospective study with external validation
Source: Front Public Health. 2026 May 19;14:1813347. doi: 10.3389/fpubh.2026.1813347 (PMC13226498; doi:10.3389/fpubh.2026.1813347)
Supplement: Supplementary file 3 [file Table_2.docx]

Supplementary Table S2  Detailed calibration and overall performance metrics of the prediction model across the training, testing, and external validation cohorts

| **Performance Metrics** | **Training Cohort** | **Testing Cohort** | **Validation Cohort** |
| --- | --- | --- | --- |
| **Overall Performance** |  |  |  |
| Brier Score (95% CI) | 0.12 (0.10–0.15) | 0.14 (0.11–0.18) | 0.15 (0.11–0.20) |
| **Calibration Metrics** |  |  |  |
| Calibration Intercept (95% CI) | 0.00 (-0.12–0.12) | 0.05 (-0.15–0.28) | -0.10 (-0.35–0.18) |
| Calibration Slope (95% CI) | 1.00 (0.88–1.12) | 0.98 (0.81–1.18) | 1.04 (0.85–1.24) |
