# Supplementary material for: Development and validation of a prediction model for infection in chronic nonhealing wounds: a two-center retrospective study with external validation
Source: Front Public Health. 2026 May 19;14:1813347. doi: 10.3389/fpubh.2026.1813347 (PMC13226498; doi:10.3389/fpubh.2026.1813347)
Supplement: Supplementary file 4 [file Table_3.docx]

**Supplementary Table S3.** Final hyperparameter configurations and optimization strategy for the optimal Random Forest model.

| **Hyperparameter (Parameter Name)** | **Description** | **Final Optimal Setting** |
| --- | --- | --- |
| **Number of trees** | The number of trees in the forest. | [e.g., 500] |
| **max_depth** | The maximum depth of the tree. Controls overfitting. | [e.g., 5] |
| **mtry** | The number of features to consider when looking for the best split. | [e.g., "sqrt" or 3] |
| **min_samples_split** | The minimum number of samples required to split an internal node. | [e.g., 10] |
| **min_samples_leaf** | The minimum number of samples required to be at a leaf node. | [e.g., 5] |
| **criterion** | The function to measure the quality of a split. | [e.g., "gini"] |
