# Supplementary material for: Development and validation of a prediction model for infection in chronic nonhealing wounds: a two-center retrospective study with external validation
Source: Front Public Health. 2026 May 19;14:1813347. doi: 10.3389/fpubh.2026.1813347 (PMC13226498; doi:10.3389/fpubh.2026.1813347)
Supplement: Supplementary file 5 [file Table_4.docx]

**Supplementary Table S4.** Sensitivity analysis comparing the predictive performance of models using binarized versus continuous representations of inflammatory markers

| **Performance Metric** | **Primary Model (Binarized Variables)** | **Sensitivity Model (Continuous Variables)** |
| --- | --- | --- |
| **Testing Cohort** |  |  |
| AUROC (95% CI) | 0.884 (0.841–0.928) | 0.895 (0.852–0.935) |
| Brier Score | 0.14 | 0.13 |
| **External Validation Cohort** |  |  |
| AUROC (95% CI) | 0.855 (0.807–0.904) | 0.862 (0.795–0.930) |
| Brier Score | 0.15 | 0.14 |
