# Supplementary material for: Development and validation of a prediction model for infection in chronic nonhealing wounds: a two-center retrospective study with external validation
Source: Front Public Health. 2026 May 19;14:1813347. doi: 10.3389/fpubh.2026.1813347 (PMC13226498; doi:10.3389/fpubh.2026.1813347)
Supplement: Supplementary file 6 [file Table_5.docx]

**Supplementary Table S5:** Descriptive performance summary of the model across major wound categories in the external validation cohort

| **Subgroup (Wound Type)** | **N** | **Infection Events, n (%)** | **AUC (95% CI)** | **Calibration (H-L test P-value)** |
| --- | --- | --- | --- | --- |
| **Diabetic Foot Ulcers** | 145 | 29 (20.0%) | 0.841 (0.762-0.920) | 0.54 |
| **Pressure Ulcers** | 100 | 15 (15.0%) | 0.865 (0.748-0.961) | 0.38 |
| **Other Wounds** | 55 | 7 (12.7%) | 0.823 (0.654-0.975) | 0.71 |

*Note: Due to the limited sample sizes and small number of events in specific subgroups, the confidence intervals are notably wide. These metrics are provided as exploratory empirical evidence of model stability and should be interpreted cautiously.
